# Open access efforts begin to bloom: *ESC Heart Failure* gets full attention and first impact factor

**DOI:** 10.1002/ehf2.12540

**Published:** 2019-10-28

**Authors:** Stefan D. Anker, Stephan von Haehling, Zoltan Papp

**Affiliations:** ^1^ Division of Cardiology and Metabolism, Department of Cardiology Charité, Campus Virchow‐Klinikum Augustenburger Platz 1 D‐13353 Berlin Germany; ^2^ Berlin‐Brandenburg Center for Regenerative Therapies (BCRT) Berlin Germany; ^3^ German Centre for Cardiovascular Research (DZHK) Berlin Germany; ^4^ Department of Cardiology and Pneumology, Heart Center Göttingen University of Göttingen Medical Center, Georg‐August‐University Göttingen Germany; ^5^ German Centre for Cardiovascular Medicine (DZHK) Göttingen Germany; ^6^ Division of Clinical Physiology, Department of Cardiology, Faculty of Medicine University of Debrecen Debrecen Hungary

## Abstract

In 2014, the Heart Failure Association (HFA) of the European Society of Cardiology (ESC) founded the first open access journal focusing on heart failure, called ESC Heart Failure (ESC‐HF). In the first 5 years, in ESC‐HF we published more than 450 articles. Through ESC‐HF, the HFA gives room for heart failure research output from around the world. A transfer process from the European Journal of Heart Failure to ESC‐HF has also been installed. As a consequence, in 2018 ESC‐HF received 289 submissions, and published 148 items (acceptance rate 51%). The journal is listed in Scopus since 2014 and on the PubMed website since 2015. In 2019, we received our first impact factor from ISI Web of Knowledge / Thomson‐Reuters, which is 3.407 for 2018. This report reviews which papers get best cited. Not surprisingly, many of the best cited papers are reviews and facts & numbers mini reviews, but original research is also well cited.

Does the world need an open access journal focusing on heart failure, some asked in 2014 when *ESC Heart Failure* (for short: ESC‐HF) had just been launched during the annual congress of the Heart Failure Association (HFA) of the European Society of Cardiology (ESC). Today, we are looking back at five years of activity with more than 450 published reviews, editorials, facts & numbers mini reviews, letters, and, most importantly, original research articles. In the first year, it was not always easy to fill the journal, but today, this is no longer an issue. In 2018, we have received 289 submissions and published 148 items—an acceptance rate of just above 50%.

In 2015, our publishers *Wiley* and we managed to get ESC‐HF listed on the PubMed website. This helped our visibility and resulted in a strong increase in submissions following this. For the aficionados of these processes, we know that only a Medline acceptance truly guarantees a full PubMed listing—but our Medline application (submitted March 2017) was successful, and full MEDLINE indexing started with the 2017 volume.

Besides PubMed, one can find ESC‐HF articles via Google Scholar since 2014. ESC‐HF is also listed in Scopus since 2014 (see http://www.scopus.com—this is the search system of *Elsevier*). In the summer of 2018, we received confirmation of our acceptance by ISI Web of Knowledge (Thomson‐Reuters) to be considered for assessment of citations and impact factor retrospectively since the start of the journal for their 2018 Journal Citation Report to come out mid‐2019. This is where things got interesting and several people performed analyses as to what our impact factor will be. At the HFA congress, we mentioned that it should be at least 3.3—we can now say that it is 3.407 for 2018. We are listed in the field ‘Cardiac & Cardiovascular Systems’ at position 49 of 136. This is a great success for the HFA and the editorial board together with our reviewers and for all of the heart failure research community. We have established an open access journal in the field of heart failure research. A very big thank you to all of you who contribute to the journal's progress and success and of course also to our many readers for whom we are doing this.

For the aficionados of technical details, we also would like to give you the details as to how our impact factor came about. The Impact Factor 2018 is calculated based on the citations in 2018 to items published in ESC‐HF in the two years before that, that is, in 2016 and 2017. This number (which is 402) is then divided by the sum of full articles (i.e. reviews and original papers) published in 2016/2017. In 2016, we published 35 full papers, and in 2017, there were 83 papers. The sum of these two years was 118, and considering the above mentioned 402 citations to these papers in the literature in 2018, our impact factor was calculated to be 3.407.

As an indication of which papers published in ESC‐HF have received most citations, we include a table that is based on ISI Web of Knowledge, as assessed on 3 August 2019, to show all articles published in 2016–2018 in ESC‐HF with >5 citations so far. Not surprisingly, many of the best cited papers are reviews and facts & numbers mini reviews, but original research is also well cited. (*Table* 1).

**Table 1:** List of all 41 articles published 2016–2018 in ESC Heart Failure with >5 citations so far

No.	First author	Title	Type	Times cited	Reference
2016	Jujo K	Randomized pilot trial comparing tolvaptan with furosemide on renal and neurohumoral effects in acute heart failure	Original research articles	29	1
2016	Konishi M	Heart failure epidemiology and novel treatments in Japan: facts and numbers	Editorials	23	2
2017	Springer J	Muscle wasting and sarcopenia in heart failure and beyond: update 2017	Reviews	21	3
2016	Nagarajan V	Obesity paradox in heart failure: a heavy matter	Reviews	16	4
2016	Arrigo M	Effect of precipitating factors of acute heart failure on readmission and long‐term mortality	Original research articles	16	5
2016	Nunez J	Left ventricular ejection fraction recovery in patients with heart failure treated with intravenous iron: a pilot study	Short communication	16	6
2017	Luedde M	Heart failure is associated with depletion of core intestinal microbiota	Original research articles	16	7
2017	Riley JP	Palliative care in heart failure: facts and numbers	Editorial	15	8
2017	Saitoh M	Anorexia, functional capacity, and clinical outcome in patients with chronic heart failure: results from the studies investigating co‐morbidities aggravating heart failure (SICA‐HF)	Original research articles	15	9
2016	Sotiropoulos K	Red cell distribution width and mortality in acute heart failure patients with preserved and reduced ejection fraction	Original research articles	14	10
2017	Delepaul B	Who are patients classified within the new terminology of heart failure from the 2016 ESC guidelines?	Original research articles	14	11
2016	Hayashi T	Subclinical hypothyroidism is an independent predictor of adverse cardiovascular outcomes in patients with acute decompensated heart failure	Original research articles	13	12
2017	Barkhudaryan A	Cardiac muscle wasting in individuals with cancer cachexia	Original research articles	11	13
2016	Sato A	Associations of dipeptidyl peptidase‐4 inhibitors with mortality in hospitalized heart failure patients with diabetes mellitus	Original research articles	10	14
2017	Amin A	On admission serum sodium and uric acid levels predict 30 day rehospitalization or death in patients with acute decompensated heart failure	Original research articles	10	15
2016	Yoshihisa A	The CHA(2)S(2)‐VASC score as a predictor of high mortality in hospitalized heart failure patients	Original research articles	9	16
2016	Hoshida S	Age‐ and sex‐related differences in diastolic function and cardiac dimensions in a hypertensive population	Original research articles	9	17
2017	Morishita T	Association between matrix metalloproteinase‐9 and worsening heart failure events in patients with chronic heart failure	Original research articles	9	18
2017	Alma LJ	Shared biomarkers between female diastolic heart failure and pre‐eclampsia: a systematic review and meta‐analysis	Review	9	19
2017	Trippel TD	Ghrelin and hormonal markers under exercise training in patients with heart failure with preserved ejection fraction: results from the EX‐DHF pilot study	Original research articles	9	20
2017	Khan MS	Renin‐angiotensin blockade in heart failure with preserved ejection fraction: a systematic review and meta‐analysis	Reviews	8	21
2017	Moeckel M	The role of procalcitonin in acute heart failure patients	Review	8	22
2018	Seropian IM	Neutrophil‐to‐lymphocyte ratio and platelet‐to‐lymphocyte ratio as predictors of survival after heart transplantation	Original research articles	8	23
2016	Searle J	Acute heart failure facts and numbers: acute heart failure populations	Editorial	7	24
2016	Thomsen MM	Varying effects of recommended treatments for heart failure with reduced ejection fraction: meta‐analysis of randomized controlled trials in the ESC and ACCF/AHA guidelines	Reviews	7	25
2016	Savage HO	Development and validation of a novel non‐contact monitor of nocturnal respiration for identifying sleep‐disordered breathing in patients with heart failure	Original research articles	7	26
2016	Aleksova N	Barriers to goals of care discussions with hospitalized patients with advanced heart failure: feasibility and performance of a novel questionnaire	Original research articles	7	27
2017	Toma M	Differentiating heart failure phenotypes using sex‐specific transcriptomic and proteomic biomarker panels	Original research articles	7	28
2017	Yoshihisa A	Associations between acylcarnitine to free carnitine ratio and adverse prognosis in heart failure patients with reduced or preserved ejection fraction	Short communications	7	29
2017	Jaarsma T	Sexual function of patients with heart failure: facts and numbers	Editorial	7	30
2017	Theidel U	Budget impact of intravenous iron therapy with ferric carboxymaltose in patients with chronic heart failure and iron deficiency in Germany	Original research articles	7	31
2018	Oehman J	Focused echocardiography and lung ultrasound protocol for guiding treatment in acute heart failure	Original research articles	7	32
2018	Pascual‐Figal D	Rationale and design of transition: a randomized trial of pre‐discharge vs. post‐discharge initiation of sacubitril/valsartan	Original research articles	7	33
2016	Meems LMG	Plasma calcidiol, calcitriol, and parathyroid hormone and risk of new onset heart failure in a population‐based cohort study	Original research articles	6	34
2016	Ahmed MB	Higher risk for incident heart failure and cardiovascular mortality among community‐dwelling octogenarians without pneumococcal vaccination	Original research articles	6	35
2016	Yamamoto E	The clinical significance of plasma neopterin in heart failure with preserved left ventricular ejection fraction	Short communications	6	36
2017	Pose A	Benefit of tolvaptan in the management of hyponatraemia in patients with diuretic‐refractory congestive heart failure: the SEMI‐SEC project	Original research articles	6	37
2017	Silvetti S	Rehospitalization after intermittent levosimendan treatment in advanced heart failure patients: a meta‐analysis of randomized trials	Original research articles	6	38
2017	Ancion A	Serum albumin level and hospital mortality in acute non‐ischemic heart failure	Original research articles	6	39
2017	Cohen‐Solal A	Beta blocker dose and markers of sympathetic activation in heart failure patients: interrelationships and prognostic significance	Original research articles	6	40
2017	Salem K	Gender‐adjusted and age‐adjusted economic inpatient burden of congestive heart failure: cost and disability‐adjusted life‐year analysis	Original research articles	6	41
2017	Jain A	The renal‐cardiac connection in subjects with preserved ejection fraction: a population based study	Original research articles	6	42
2017	Lancellotti P	Protocol update and preliminary results of EACVI/HFA Cardiac Oncology Toxicity (COT) Registry of the European Society of Cardiology	ESC and HFA paper	6	43
2017	Peled Y	The impact of gender mismatching on early and late outcomes following heart transplantation	Original research articles	6	44
2017	Koyama S	Dynamic changes of serum microRNA‐122‐5p through therapeutic courses indicates amelioration of acute liver injury accompanied by acute cardiac decompensation	Original research articles	6	45
2018	Porto C	Association between vitamin D deficiency and heart failure risk in the elderly	Original research articles	6	46
2018	Ferreira JP	Rationale of the fibrotargets study designed to identify novel biomarkers of myocardial fibrosis	Original research articles	6	47
2018	Mustroph J	Empagliflozin reduces Ca/calmodulin‐dependent kinase II activity in isolated ventricular cardiomyocytes	Short communication	6	48
2018	Smedema JP	Right ventricular involvement and the extent of left ventricular enhancement with magnetic resonance predict adverse outcome in pulmonary sarcoidosis	Original research articles	6	49
2018	Yoshihisa A	Liver fibrosis score predicts mortality in heart failure patients with preserved ejection fraction	Original research articles	6	50

There is a lot of work where people say that nothing is really wrong with it. It may just be a little small in study size, lacking the extra control group or extra biomarker tests that some would have hoped for or that simply, for these and many other reasons, may not have priority for the limited publication space in the premier cardiology and heart failure specialist journals. We want to give room for the many other good pieces of heart failure research from around the world. This is also why we have installed a transfer process from the *European Journal of Heart Failure* to ESC‐HF, which is now the source of half of all submissions to our journal.

To manage the increasing number of submissions while maintaining a reasonable time from submission to review decision and Early View publication, we have substantially increased the size of the editorial board. If some of you feel qualified and are interested to help in this, please let us know. The biggest problem we face for a timely publication process is finding reviewers for your submissions. As many have asked in this context, we do not mind if you inform us about previous reviewer comments in your submission cover letters, but we then also request that you add response comments in your submission package for us to consider. But of course, we will trigger also our own reviewer activities. And for this, a longer rather than shorter list of suggested reviewers would also be very helpful if you want to technically support your submission to ESC‐HF.

We hope that together we can keep the journal at a good quality standard, even if more papers are published in the future. We strongly suspect that submissions will further increase. From 2018 to 2019, the projected growth in submissions is >30%. And we apologize already, if sometimes there are still some delays. We do our best with the staff in Berlin and Göttingen and Debrecen. And if you sometimes feel that we are like the PLoS‐ONE of heart failure, then indeed this is what we wish to be, the major publication threshold being that the work under consideration is ‘scientifically sound’. But there are two major differences, which is that we have the support of a strong society and that a good proportion of the papers we accept also comes out in print.

At last, some also have asked us about our plans and ambitions for future impact factors of ESC‐HF. We are not striving to get the top of heart failure research—we know that they primarily published elsewhere. We are aiming for an impact factor above 4, but we are committed to essentially unlimited growth of the journal. We are aiming for an acceptance rate of 40–50%. We believe that the main job of a journal like ours is to give room for discussion and for any interesting research. We are aiming to get your research published, let you keep the copyright, and make the research available for the whole world to read at no cost. This is the open access model at work for heart failure. This is *ESC Heart Failure* for you.
